# Role of HGF in epithelial–stromal cell interactions during progression from benign breast disease to ductal carcinoma *in situ*

**DOI:** 10.1186/bcr3476

**Published:** 2013-09-12

**Authors:** Patricia Casbas-Hernandez, Monica D’Arcy, Erick Roman-Perez, Heather Ann Brauer, Kirk McNaughton, Samantha M Miller, Raghav K Chhetri, Amy L Oldenburg, Jodie M Fleming, Keith D Amos, Liza Makowski, Melissa A Troester

**Affiliations:** 1 Department of Pathology and Laboratory Medicine, School of Medicine, University of North Carolina at Chapel Hill, Chapel Hill, NC 27599, USA; 2 Department of Epidemiology, Gillings School of Global Public Health, University of North Carolina at Chapel Hill, Chapel Hill, NC 27599, USA; 3 Department of Cell and Molecular Physiology School of Medicine, University of North Carolina at Chapel Hill, Chapel Hill, NC 27599, USA; 4 Department of Physics and Astronomy, University of North Carolina at Chapel Hill, Chapel Hill, NC 27599, USA; 5 Department of Biology, North Carolina Central University, Durham, NC 27707, USA; 6 Department of Surgery, Biomedical Research Imaging Center, University of North Carolina School of Medicine, Chapel Hill, NC 27599, USA; 7 Lineberger Comprehensive Cancer Center, University of North Carolina at Chapel Hill, Chapel Hill, NC 27599, USA; 8 Department of Nutrition, Gillings School of Global Public Health, University of North Carolina at Chapel Hill, Chapel Hill, NC 27599, USA

## Abstract

**Introduction:**

Basal-like and luminal breast cancers have distinct stromal–epithelial interactions, which play a role in progression to invasive cancer. However, little is known about how stromal–epithelial interactions evolve in benign and pre-invasive lesions.

**Methods:**

To study epithelial–stromal interactions in basal-like breast cancer progression, we cocultured reduction mammoplasty fibroblasts with the isogenic MCF10 series of cell lines (representing benign/normal, atypical hyperplasia, and ductal carcinoma *in situ*). We used gene expression microarrays to identify pathways induced by coculture in premalignant cells (MCF10DCIS) compared with normal and benign cells (MCF10A and MCF10AT1). Relevant pathways were then evaluated *in vivo* for associations with basal-like subtype and were targeted *in vitro* to evaluate effects on morphogenesis.

**Results:**

Our results show that premalignant MCF10DCIS cells express characteristic gene expression patterns of invasive basal-like microenvironments. Furthermore, while hepatocyte growth factor (HGF) secretion is upregulated (relative to normal, MCF10A levels) when fibroblasts are cocultured with either atypical (MCF10AT1) or premalignant (MCF10DCIS) cells, only MCF10DCIS cells upregulated the HGF receptor MET. In three-dimensional cultures, upregulation of HGF/MET in MCF10DCIS cells induced morphological changes suggestive of invasive potential, and these changes were reversed by antibody-based blocking of HGF signaling. These results are relevant to *in vivo* progression because high expression of a novel MCF10DCIS-derived HGF signature was correlated with the basal-like subtype, with approximately 86% of basal-like cancers highly expressing the HGF signature, and because high expression of HGF signature was associated with poor survival.

**Conclusions:**

Coordinated and complementary changes in HGF/MET expression occur in epithelium and stroma during progression of pre-invasive basal-like lesions. These results suggest that targeting stroma-derived HGF signaling in early carcinogenesis may block progression of basal-like precursor lesions.

## Introduction

Normal development and homeostasis requires epithelial–stromal interactions. Cancers must evolve and adapt in stromal context, and therefore cancer progression depends on an initiated cell’s ability to utilize permissive signals and circumvent repressive signals [1]. Under evolutionary theories of cancer, tumors that progress have characteristics that are advantageous given their microenvironments [2]. Cancer cells may also modify their environments to induce growth-promoting signals. Recent data suggest that host and/or stromal factors affect the tumor subtype. For example, aging stroma may influence which tumor subtypes develop or may promote more aggressive disease [3,4]. Conversely, tumor characteristics may define epithelium–stromal interactions. Basal-like breast cancers have a distinct microenvironment interaction pattern relative to other breast cancer subtypes [5] and appear to be associated with distinct immune microenvironments [6-8]. These and many other data suggest that complementary epithelial–stromal coevolution is influential in cancer development. However, since most of these studies have examined epithelial–stroma interactions after tumors have acquired invasive characteristics, it is not well known how host–tumor interactions are maintained earlier in disease progression.

We hypothesized that basal-like breast cancers may have unique interactions with their microenvironments beginning in the early stages of progression. In epidemiologic studies, there is evidence that basal-like breast cancers progress very rapidly through the ductal carcinoma *in situ* (DCIS) stage compared with other cancers [9]. However, many of the DCIS-adjacent stromal tissue studies have been from patients who also have invasive cancers in the same breast [10], and given the cross-sectional nature of these studies (with data at only a single time point in the progression of disease) it is difficult to identify epithelial–stromal interactions that are induced during progression. In addition, stroma from DCIS lesions and invasive tumors are very similar, suggesting that stromal changes may occur prior to invasion [10,11]. It is important to identify pathways that are altered in the stroma prior to invasion as these pathways may be targetable.

To study epithelial–stromal interactions in the pre-invasive phases of basal-like breast cancer development, we employed the MCF10 cell line series in cocultures. The MCF10 cell lines represent an isogenic background (being derived from a single patient), but express pathologic characteristics in xenografts, ranging from non-neoplastic benign morphology (MCF10A) to atypical hyperplasia (MCF10AT1) to DCIS (MCF10DCIS). These lines were cocultured with fibroblasts (both two-dimensional on plastic and three-dimensional (3D) in Matrigel®/collagen). Cell-based assays and gene expression profiling were conducted to track the evolution of cell–cell interactions with progression. The resulting experimental data, together with patient data, suggest an important role for hepatocyte growth factor (HGF) signaling in premalignant to invasive basal-like breast cancer.

## Methods

### Cell lines and treatments

MCF10A, MCF10AT1 and MCF10DCIS.com (referred to as MCF10DCIS) were purchased from Karmanos Cancer Institute (Detroit, MI, USA) and Asterand (Detroit, MI, USA). These cell lines were maintained in Dulbecco’s modified Eagle’s medium supplemented with 5% horse serum, 50 units/ml penicillin, and 50 units/ml streptomycin, 5 μg/ml insulin (GIBCO, Life Technologies, Carlsbad, CA, USA), 1 μg/ml hydrocortisone (Sigma-Aldrich, St. Louis, MO, USA), cholera toxin (EMD Millipore, Darmstadt, Germany) and Epidermal Growth Factor (Invitrogen, Life Technologies). Cocultures were also performed in this media after ascertaining that reduction mammoplasty fibroblasts (RMFs) maintained their RPMI 1640 doubling times in this Dulbecco’s modified Eagle’s medium/F12.

MCF7 cells (luminal cell line) and SUM149 cells (basal-like cell line) were purchased from ATCC (Manassas, VA, USA). RMFs (htert-immortalized fibroblasts from reduction mammoplasty [12]) were provided by Dr Charlotte Kupperwasser (Tufts University, Medford, MA, USA). Similar to previous studies [13], we selected an htert-immortalized fibroblast cell line over primary cell lines for several reasons. Primary cells have a limited lifespan; after nine passages they senesce, allowing insufficient time to perform many assays (such as the 3D assays described below). Even prior to senescence, the aging process and patient-to-patient variation affects gene expression, as shown by quantitative reverse transcription PCR in Additional file 1. Primary fibroblasts were obtained from breast tissue of patients undergoing breast surgery for primary invasive breast carcinoma at UNC Hospitals. Tissue specimens were procured under an Institutional Review Board-approved protocol by the Lineberger Cancer Center Tissue Procurement Facility. The isolation protocol of these cancer- adjacent normal-associated fibroblasts and cancer-associated fibroblasts was described previously [13]. For this panel of 14 primary cancer-normal associated fibroblasts (obtained from histologically normal tissue adjacent to a tumor) and cancer-associated fibroblasts at different passages, HGF levels vary by more than 500-fold. These cell lines were maintained at 37°C and 5% CO_2_ in RPMI 1640 or Dulbecco’s modified Eagle’s medium/F12 with l-glutamine (GIBCO) supplemented with 10% fetal bovine serum (Sigma-Aldrich) and 50 units/ml penicillin and 50 units/ml streptomycin (GIBCO) as described elsewhere [5,13]. All cell lines were tested for mycoplasma prior to use by the University of North Carolina at Chapel Hill Tissue Culture Facility.

### Coculture conditions and treatments

Two types of cocultures were performed using media and culture conditions described above and previously [5]. Direct cocultures are defined as a coculture where the two cell types are grown in direct physical contact, in the same well. The epithelial:RMF ratios plated for these cocultures were 1:4, 1:2, 1:1 and 2:1 and these cocultures, as well as the monocultures of each cell line, were maintained for 48 hours before RNA isolation.

Indirect cocultures are defined as a coculture where the fibroblasts and cancer cells are separated by a porous membrane that allows cell–cell communication via soluble factors. For indirect cultures, fibroblasts were seeded on inserts on Corning (Tewksbury, MA, USA) Transwell plates with 0.4 μm-pore polycarbonate membranes; epithelial cells were grown in the bottom well. Indirect cultures were plated at a 1:1 ratio and maintained for 48 hours before RNA isolation.

### RNA and expression microarrays

Cells were harvested by scraping in RNA lysis buffer. Total RNA was isolated using the RNeasy mini kit (Qiagen, Valencia, CA, USA) and RNA quantification was performed on a ND-1000 Nanodrop spectrophotometer and RNA quality was analyzed on an Agilent 2100 Bioanalyzer using a RNA6000 nano chip (Agilent, Santa Clara, CA, USA). Microarrays were performed according to the Agilent protocol using two-color Agilent 4×44K V2 (Agilent G4845A). We used the Agilent Quick Amp labeling kit and protocol to synthesize Cy3-labeled reference from Stratagene Universal Human Reference spiked at 1:1,000 with MCF7 RNA and 1:1,000 with ME16C RNA to increase expression of breast cancer genes. The identical protocol was applied to total RNA from cocultured or monocultured cell lines to label these samples with Cy5. Labeled cDNAs were hybridized to arrays overnight and washed before scanning on an Agilent G2505C microarray scanner.

### Coculture data normalization and analysis

Data from 47 microarrays (representing monocultures, direct cocultures, and indirect cocultures from six different cell lines) were included in this study. Microarray data are available in the Gene Expression Omnibus [GSE43467]. Only those genes where more than 70% of microarrays had signals in both channels greater than 10 dpi were included. Data were Lowess normalized and missing data were imputed using *k*-nearest neighbors’ imputation.

For the direct coculture analyses, we excluded genes that did not have at least twofold deviation from the mean in at least one sample and the method of Buess and colleagues [14] was used to normalize cocultures to appropriate monocultures performed in the same media and under identical conditions as described previously [5]. Briefly, the Buess method is an example of an expression deconvolution approach applied to coculture data; this method estimates the percent of fibroblasts and cancer cells in each coculture, and normalizes the data for composition differences prior to estimating the effect of epithelial–stromal interaction on gene expression. The Buess interaction coefficient *I* was calculated as the ratio of observed to expected gene expression and an *I*-matrix representing the epithelial–stromal interaction coefficient for each gene in each coculture was generated. The estimated *I* value for each gene and coculture can be thought of as an indicator of the ratio of that gene’s expression level relative to the expected level based on the cellular composition and the monoculture expression values. For coculture studies, *I*-matrices were analyzed using multiclass significance analysis of microarrays [15], comparing MCF10A cocultures with MCF10AT cocultures with MCF10DCIS cocultures (three classes).

Microarray analysis was carried out using R version 1.14. Heatmap generation and visualization were performed using Cluster 3.0 and Java treeview, respectively. Functional and pathway analyses were carried out using ingenuity pathway analysis (IPA, Redwood City, CA, USA), with Benjamini–Hochberg multiple testing correction to identify significant functions and pathways with *P* <0.05. Pathways and functions with two or fewer genes were excluded from our analysis.

### Calculation of basal-like interaction score

We utilized gene sets identified in Camp and colleagues [5] to score each coculture for the degree to which it expressed basal-like microenvironment genes. In Camp and colleagues’ study a 30-gene signature was identified that predicted basal-like versus luminal interactions in cocultures and that also distinguished basal-like versus luminal tumors. Using *I* values (as described above) for each of these 30 genes across all cocultures, we computed a basal-like interaction score. Briefly, using the method of Creighton and colleagues [16], vectors corresponding to the 30 genes in the basal-like signature were constructed, with 1 assigned to genes upregulated in basal-like cocultures/cancers and −1 assigned to downregulated genes. A Pearson correlation coefficient was calculated for this standard vector versus the vector of *I* values for each coculture experiment. The Pearson correlation coefficient from this analysis is defined as the basal-like interaction score.

### Analysis of cytokine expression in conditioned media (cytokine array)

To identify soluble mediators of basal-like microenvironments in the MCF10DCIS cells, conditioned media samples from direct 1:1 cocultures (48 hours, standard coculture media conditions as described above) were analyzed according to manufacturer protocol on a RayBio Human Cytokine Antibody Array 5 (80) (Raybiotech, Norcross GA, USA) designed to detect 80 cytokines and chemokines. Briefly, slides were blocked by incubation with blocking buffer at room temperature for 30 minutes and incubated with 100 μl conditioned media at room temperature for 90 minutes. Slides were washed and incubated with biotin-conjugated antibodies overnight at 4°C. Finally, the slides were washed and incubated with fluorescent dye-conjugated streptavidin at room temperature for 2 hours. After final washing, slides were dried by centrifugation at 0.2 RCF (Centrifuge 5702R; Eppendorf, Hauppauge, NY, USA) for 3 minutes. The fluorescent signal was detected on a laser scanner (Axon, Molecular Devices, LLC, Sunnyvale, CA, USA) using a Cy3 (green) channel (excitation frequency 532 nm). Data for each cytokine were normalized to positive controls on the same slide to estimate relative protein expression. Each monoculture or direct coculture was analyzed in duplicate.

### Western blot

Cells were harvested from interaction cocultures and protein was isolated and quantified as described previously [17]. Lysates were denatured by boiling with β-mercaptoethanol, and 15 to 30 μg protein were electrophoresed on a 4 to 20% Tris–HCl Criterion precast gel (Bio-Rad, Hercules, CA, USA) and transferred to a Hybond-P membrane (Amersham-GE Healthcare Life Sciences, Pittsburgh, PA, USA) by electroblotting. The blots were probed with antibodies against the receptor MET (#8198S; Cell Signaling, Beverly, MA, USA), HGFα chain (sc-166724; Santa Cruz, Dallas, TX, USA) and β-actin (#4967; Cell Signaling, Beverly, MA, USA). Blots were washed three times with Tris-buffered saline supplemented with 0.1% TWEEN and then were probed with ECL anti-mouse IgG horseradish peroxidase-linked whole antibody from sheep (Amersham-GE Healthcare, Life Science). Blots were rewashed, and detection was by enhanced chemiluminescence western blotting detection system (Amersham-GE Healthcare Life Science). Relative MET and HGF protein concentrations were quantified using ImageJ software, with the pixel intensity of the MET or HGF protein band divided by the pixel intensity of the β-actin band. Fold-change expression was calculated by dividing the coculture expression by the monoculture expression at that same time point.

### Quantitative PCR for MET and HGF

The relative quantity of HGF (Hs00300159_m1, ABI catalogue number 4331182) and MET (Hs01565584_m1, ABI catalogue number 4331182) mRNA was assayed by quantitative PCR using an ABI 7900HT machine (Life Technologies, Carlsbad, CA, USA). mRNA was isolated from cells using Qiagen’s RNeasy mini kit and protocols (Qiagen, Valencia, CA, USA). Then 1 μg total RNA was reverse transcribed into cDNA using the iScript cDNA synthesis kit and protocol from Bio-Rad. The cDNA was then diluted fivefold by the addition of 80 μl water. Subsequently, 2 μl cDNA and 18 μl master mix 10 μl SsoFast 2X Probes Supermix (Bio-Rad), 0.5 μl 18S-VIC and 0.5 μl gene-specific Assay-On-Demand-FAM (ABI Life Technologies, Grand Island, NY, USA), 7 μl water were used in each well of the quantitative PCR 96-well plate. Amplification conditions were as follows: one cycle of 95°C for 1 minute, 40 cycles of 95°C for 5 seconds, and 60°C for 20 seconds.

### Generation of coculture-derived HGF signature

To identify HGF-regulated genes, monocultures of MCF10DCIS cells were grown in serum-free media and treated with recombinant human HGF (294-HG/CF; R&D Systems, Minneapolis, MN, USA) for 6 hours with addition of HGF every hour at a 100 ng/ml concentration (half-life of HGF is approximately 4 minutes). Total RNA was isolated after 6 hours of treatment and analogous cocultures with fibroblasts were performed. Microarray data from both HGF-treated and fibroblast-cocultured cell lines were normalized to sham monocultures (monocultures in serum-free media with no recombinant human HGF and monocultures in regular media) by subtracting the log_2_(R/G) values of the monoculture. The resulting log_2_(R/G) ratio represents the response to coculture or treatment relative to that of sham. To identify genes that were differentially regulated by both coculture and HGF treatment, a one-class significance analysis of microarrays was performed with all HGF-treated and cocultured arrays first normalized to sham monocultures. Functional and pathway analyses of the resulting gene signatures were performed <less than 0.05 after Benjamini–Hochberg multiple testing correction.

### Correlation with HGF signature in human tumors

We evaluated the behavior of our HGF signature (described above) in 707 breast cancer samples from three publically available datasets: NKI295 (*N* = 295) [18], Naderi and colleagues (*N* = 135) [19], and UNC337 samples (*N* = 277) [20]. First, intrinsic subtype classification was performed using the PAM50 predictor of Parker and colleagues [21]. Next, expression of the HGF signature was evaluated by mapping the 280-gene HGF signature to all three datasets. A common probe set representing 109 unique genes was identified. These probes were median-centered across samples, and then duplicate genes were collapsed to a unique entry ID by statistical mean. Tumors were classified as HGF-positive or HGF-negative using methods described above and in Creighton and colleagues [16]. Correlations >0 were classified as positive for the HGF signature (HGF-positive) and correlations ≤0 were classified as HGF-negative. We then obtained a chi-square statistic (four degrees of freedom) to test the association between tumor subtype (basal-like, luminal A, luminal B, Her2, normal-like) and HGF class (HGF-positive vs. HGF-negative). All statistical analyses were performed using R version 1.14 and Bioconductor packages.

### Three-dimensional morphogenesis assay

3D cocultures were performed as described previously [22,23]. A model system similar to that of Jedeszko and colleagues was used; however, our RMF lines are not engineered to overexpress HGF [24]. Briefly, a 1:3 ratio of epithelial:RMF cells were cocultured in a 3D extracellular scaffold composed of a 1:1 mixture of biologically derived collagen I and Matrigel® (BD Biosciences, San Jose, CA, USA). The final concentration of collagen I was 1 mg/ml. One well from a 24-well plate was prepped for coculture by coating with 500 μl Matrigel®–collagen mix. Then, 1 ml cell suspension in Matrigel®–collagen mix was plated. Cultures were maintained in a humidified, 37°C, 5% CO_2_ incubator for 2 weeks, with media change every 2 days. To test the role of HGF in morphogenesis of these cocultures, a set of plates were cultured with neutralizing, anti-HGF antibody (0.5 mg/ml, Abcam10678; Abcam, San Francisco, CA, USA), which was added every day for 2 weeks using previously published protocols [13].

The Matrigel®–collagen-embedded 3D structures were fixed after 2 weeks using 4% paraformaldehyde (USB, Cleveland, OH, USA) overnight. Fixed cultures were then cryopreserved in 20% sucrose in 0.1 M phosphate buffer at 4°C and washed before embedding and freezing in optimal cutting temperature compound (Tissue-tek 4538; Miles Laboratories Inc, Naperville, IL, USA). Frozen sections (6 μm) were cut for immunohistochemistry using a Leica 1950 cryostat (Leica Biosystems, Buffalo Grove, IL, USA). For immunostaining, slides were brought to room temperature, hydrated and placed in citrate buffer pH 6.0 (TA135-HBH; Thermo-Fisher, Waltham, MA, USA). Heat-induced epitope retrieval was performed using a decloaking chamber (Biocare Medical, Concord, CA, USA) at 95°C for 5 minutes followed by 90°C for 10 seconds. Slides were cooled for 20 minutes, washed in Tris buffer (0.05 M pH 7.6) and blocked in 10% normal goat serum in Tris for 1 hour at room temperature. Samples were incubated overnight at 4°C in mouse monoclonal smooth muscle actin (M085; 1:100; Dako, Carpinteria, CA, USA) and rabbit polyclonal cytokeratin (Z0622; 1:100; Dako). After rinsing, slides were incubated at room temperature for 3 hours in a mixture containing goat anti-mouse antibody (Alexafluor 568, Invitrogen A21134; 1:400) and goat anti-rabbit antibody (Alexafluor 488, Invitrogen A11008; 1:400). Slides were washed and coverslipped with Fluorogel II containing 4′,6-diamidino-2-phenylindole (EM Sciences, Darmstadt, Germany). For hematoxylin and eosin (H&E) stain, frozen sections were stained 1 minute in acidified Harris Hematoxylin (6765003; Thermo Scientific), rinsed in running tap water for 4 minutes, stained for 3 minutes in alcoholic Eosin Y (6766007; Thermo Scientific), dehydrated in 95% alcohol for 2 minutes and for 5 minutes in absolute alcohol, cleared in xylene for 6 minutes and coverslipped with DPX mountant. Phase and fluorescent images were obtained using an Olympus (Tokyo, Japan) IX-81 microscope at 10× magnification.

Acinar morphology and apoptosis were assessed using H&E slides of 3D sections. Apoptosis was present if apoptotic bodies (small, sealed membrane vesicles) were present [25]. Immunofluorescence staining of pan-cytokeratin was used to score each organoid for lumen (present or absent) and to confirm the epithelial cell identity of acinar cells. Each structure was visualized, and classified as with lumen if there was a clear open space within the center of the structure or as no lumen if the lumen was filled by cells or cellular debris. For lumen and apoptosis, 30 to 35 acinar structures were analyzed per condition.

Lumen size and total acinar size were also measured by a method based on optical coherence tomography (OCT). Imaging of the 3D cultures was performed using a custom, ultrahigh-resolution, spectral-domain OCT system as previously described in [22]. The OCT image stacks were resampled into an isotropic pixel resolution of 1.55 μm after correcting for the refractive index of the aqueous gels, and displayed in a hot color map using MATLAB® (2011a, MathWorks, Natick, MA, USA). From color-mapped OCT images, cell clusters resembling acini were selected based on their spherical shape. The OCT image containing the central position of each acinus was determined by sifting through the OCT image-stack to find the image with the largest acinus size. The overall acinus area and acinus lumen area were each characterized from these central OCT image slices using ImageJ. The mean acini area and mean lumen area were calculated for each gel. A total of 50 to 60 acinar structures were analyzed per condition.

### Population doubling times

Cell line population doubling times (PDTs) were determined in 1:1 ratio indirect cocultures by plating 1×10^5^ cells in each well or insert of a six-well dish. Two dishes per cell line were counted at 24 and 48 hours after plating. Anti-HGF antibody (0.5 mg/ml, Abcam10678) was added every 12 hours to the cocultures. The growth of the cells during the log phase can be modeled using the following equation:

(1) $$\text{lnA}\left(t\right)=\text{ln}A_{0}=\text{kt}$$

where *A*(*t*) is the number of cells per plate at time *t*, and *k* represents the first-order rate constant of cell growth, with units d^–1^. Using this regression equation, independent estimates of *k* were obtained for MCF10A and MCF10DCIS in coculture with and without anti-HGF antibody. The PDT for each cell line in each condition was calculated as:

(2) $$\text{PDT}=\frac{0.693}{k}$$

To compare the growth rates, *k*, for two cell lines, a multiple regression model similar to that described by Troester and colleagues [26] was employed.

### Statistical analysis

Statistical analyses for experimental results were performed using SAS 9.2 (SAS Institute, Cary, NC, USA) (32). Analysis of variance was performed to compare basal-like interaction scores across MCF10A, MCF10AT1 and MCF10DCIS cocultures. Two-tailed *t* tests were performed to determine statistical differences between lumen size (by OCT measurement in 3D morphogenesis assay) of MCF10A and MCF10DCIS, with and without anti-HGF. A chi-square analysis was performed comparing the presence of apoptosis (yes/no) and lumen (yes/no) in the 3D morphogenesis in MCF10A, MCF10DCIS and MCF10DCIS + anti-HGF cocultures.

## Results

### The MCF10A series acquire basal-like microenvironment characteristics at the DCIS stage

**Figure 1:**
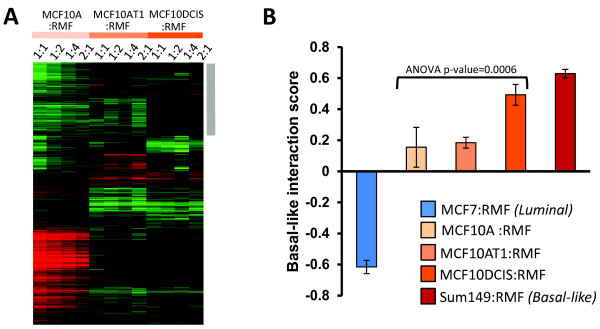
**MCF10A series progressively acquires basal-like microenvironment characteristics. (A)** One-dimensional (genes only) hierarchical clustering showing interaction scores (*I* values) for genes that significantly change due to coculture. Distinct clusters of genes are upregulated in each cell line. A gray bar adjacent to the heat map shows a cluster of genes that are uniquely upregulated in the MCF10DCIS:RMF cocultures. **(B)** A basal-like interaction score was developed previously by Camp and colleagues [5]; the coculture data show a strong relationship between progression and expression of basal-like microenvironment characteristics. Luminal cocultures had a negative basal-like interaction score, while the score increased with progression from A/AT1 to ductal carcinoma *in situ* (DCIS), with DCIS having interaction scores similar to those in the basal-like invasive cancer cell line SUM149. ANOVA, analysis of variance; RMF, reduction mammoplasty fibroblast.

Each cell line in the MCF10 isogenic panel had a distinct response to coculture with RMFs. By multiclass significance analysis of microarrays, we identified approximately 700 genes as differentially expressed across these three cell lines (Figure 1A). One set of genes was particularly upregulated in MCF10DCIS cells, and not in MCF10A or MCF10AT cells (gray bar in Figure 1A). This cluster of genes was analyzed by IPA (Additional file 2) and results suggest immune response processes and connective tissue disorders, such as immune cell trafficking (*P* = 0.021), acute phase response signaling (*P* <0.001) or cell-mediated immune response (*P* = 0.005). Many of these processes were also upregulated in invasive basal-like breast cancers in direct cocultures [5].

We utilized a gene set identified by Camp and colleagues [5] to directly test whether the cocultures upregulated basal-like microenvironment characteristics. This signature can distinguish basal-like from luminal cocultures *in vitro* and can distinguish basal-like from luminal tumors *in vivo*. Using this signature we calculated a score for each sample, termed the basal-like interaction score. MCF10DCIS cells had a high basal-like interaction score (Figure 1B), similar to that of the invasive basal-like breast cancer cell line SUM149. In contrast, the cocultures of premalignant MCF10AT1 and MCF10A cells showed weakly positive basal-like interaction scores and the luminal MCF7 cell line showed a negative score (Figure 1B). Pearson correlations were also performed for the monocultures and indirect cocultures of these same cell lines (Additional file 3). The correlation observed in the indirect cocultures followed the same trend as observed in direct cocultures, although the strength of the basal-like score was somewhat attenuated. This attenuation was expected based on dilution and diffusion of secreted signals; in indirect cocultures, signals from the fibroblasts are diluted in large volumes of media, whereas in direct coculture the dependence on diffusion kinetics and protein stability is reduced. While neither direct nor indirect cocultures simulate the inhibitory effects of basement membrane on cellular signaling [27-29], direct cocultures offer important advantages to address cell–cell signaling, particularly when considering effectors with short half-lives. Thus, all coculture data are from direct cocultures unless otherwise stated.

### Upregulation of secreted cytokines in MCF10DCIS fibroblast cocultures

**Figure 2:**
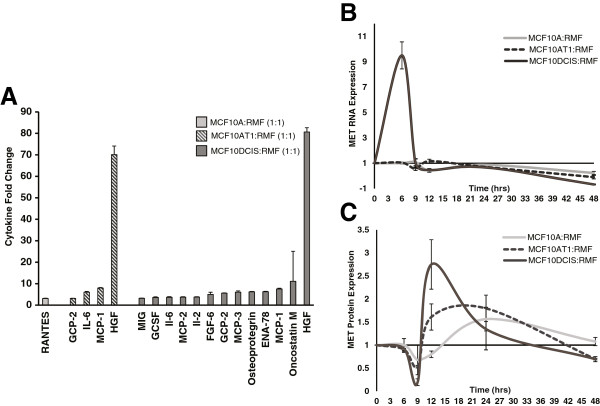
**MCF10A series cocultures present differentially secreted cytokines and MET receptor status. (A)** A variety of cytokines are overexpressed in the direct cocultures of all three cell lines. The number of cytokines overexpressed increases with progression. The cytokine most strongly overexpressed was hepatocyte growth factor (HGF). **(B)** MET RNA fold-change expression relative to the corresponding cell line in monoculture over a 48-hour period. We observe a sharp increase of transcript in the MCF10DCIS cells when in coculture after 6 hours. **(C)** MET protein fold-change expression relative to the corresponding cell line in monoculture over a 48-hour period. The protein increase is delayed 6 hours when compared with RNA expression due to the delay in translation. FGF, fibroblast growth factor; GCP, granulocyte chemotactic peptide; GCSF, granulocyte colony-stimulating factor; MCP, monocyte chemotactic peptide; MIG, monokine induced by interferon-gamma; RANTES, regulated on activation, normal T-cell expressed and secreted; RMF, reduction mammoplasty fibroblast.

Having established that basal-like microenvironments are induced by soluble factors, we sought to identify the secreted mediators. Eighty cytokines and chemokines were measured in the conditioned media of the direct cocultures. A striking increase in the number of cytokines expressed occurred in the MCF10DCIS cocultures, with a total of 62 cytokines upregulated by more than 1.5-fold. In contrast, MCF10A and MCF10AT cocultures each upregulated only a small number of cytokines (Table 1; a full list of cytokines and their fold-change relative to monoculture is provided in Additional file 4). The most highly upregulated cytokine in DCIS cocultures was HGF, which increased monotonically from MCF10A to MCF10AT1 to MCF10DCIS and was upregulated more than 80-fold in MCF10DCIS and 70-fold in MCF10AT1 direct cocultures (Figure 2A).

**Table 1:** Number of cytokines expressed in the cocultures of the MCF10A series

	**Cytokine fold-change**			
	**1.5 to 3**	**3 to 10**	**>10**	**Total number**
MCF10A:RMF	-	1	-	1
MCF10AT1:RMF	5	2	1	8
MCF10DCIS:RMF	50	11	1	62

*RMF* reduction mammoplasty fibroblast.

When HGF secretion is measured in the conditioned media from cocultures, the source of the HGF is not discernible. Therefore, to identify which component of the system is producing the HGF, intracellular HGF RNA and protein levels were measured in each cell line after 48 hours of coculture. Additional file 5 shows that HGF is exclusively being produced by the fibroblasts. The epithelial cells had no detectable levels of transcript or protein in the monoculture; however, in coculture some HGF protein was observed in the epithelial cells, presumably due to internalization of the receptor–ligand complex. HGF secretion and activation is part of a complex cascade that regulates the actions of HGF. Consistent with previously published results suggesting that activity of HGF is regulated pericellularly on the protein level by hepatocyte growth factor activator inhibitor type 1 (HAI-1) [30], we found HAI-1 differentially expressed in our heat map from Figure 1A. HAI-1 inhibits HGF activator, thereby inhibiting the activation and subsequent activity of HGF. HAI-1 was more highly expressed in MCF10A cocultures, and is associated with lower HGF secreted protein by cytokine array analysis.

HGF is the major ligand for the MET tyrosine kinase receptor and is also a negative regulator of MET transcription [31]. To evaluate whether MET is present in these cell lines, and whether MET levels are differentially regulated in coculture conditions, we assayed expression of this receptor in the MCF10 series, alone and in coculture over the course of 48 hours. Both at the RNA (Figure 2B) and the protein level (Figure 2C), we observed that contact with fibroblasts (6 hours) induced the MCF10DCIS cells to markedly upregulate MET RNA. Peak RNA induction at 6 hours is followed by peak protein expression at 12 hours. This effect was not observed (in MCF10A) or markedly diminished (in MCF10AT1) in the other two cell lines of the series. The interaction of MCF10DCIS cells with RMF in coculture thus stimulates an increase in HGF secretion and a concomitant increase in epithelial HGF receptor, MET, expression.

### An *in vitro*-generated HGF gene signature correlates with basal-like tumors

Our coculture results established a fold-change increase in HGF signaling in premalignant, basal-like microenvironments; however, if these changes are essential for basal-like breast carcinogenesis, then they should also be present in invasive basal-like breast cancers. To assess this hypothesis, we generated an *in vitro* HGF signature. We identified gene expression changes that occurred in both MCF10DCIS monocultures treated with recombinant human HGF and cocultures of MCF10DCIS with RMFs. These HGF-regulated genes are most likely to be effecting the action of HGF on MET in coculture. IPA analysis of the HGF signature suggested sonic hedgehog signaling (*P* = 0.007), basal cell carcinoma signaling (*P* = 0.017), and tight junction signaling (*P* = 0.020) among other signaling pathways were upregulated by HGF signaling in cocultures.

**Figure 3:**
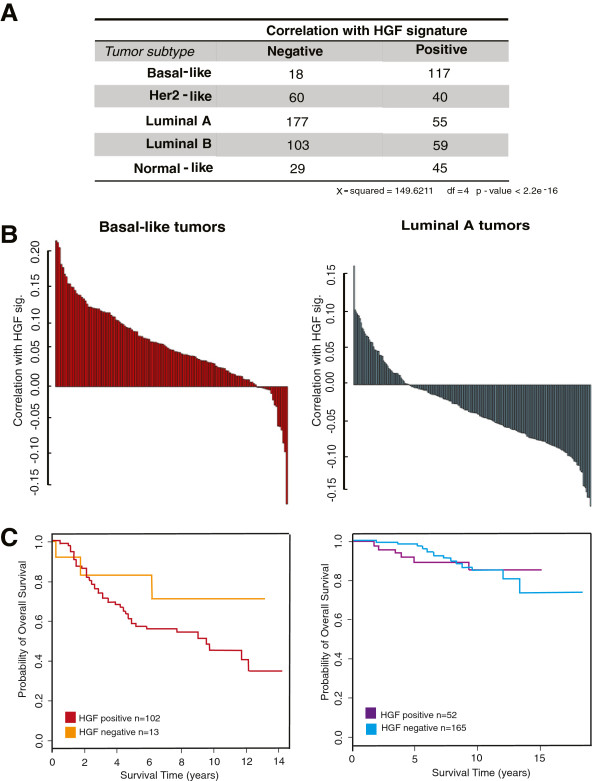
**Hepatocyte growth factor signaling is present *in vivo *in basal-like tumors.** An *in vitro* generated signature that captures hepatocyte growth factor (HGF) signaling is highly correlated with invasive basal-like tumors. **(A)** Number of tumors that are positively and negatively correlated with the HGF signature by subtype. **(B)** Creighton correlation of each basal-like and luminal tumor with the HGF signature; the degree of positivity among basal-like tumors is higher when compared with the luminal tumors (scale bar). **(C)** Kaplan–Meier survival curves for overall survival among patients that were positive or negative for the HGF signature. Patients with basal-like tumors with positive HGF signatures had worse overall survival over a period of 14 years.

Using this signature we scored 707 invasive tumors from three independent datasets as having a high or low correlation with this HGF signature. Figure 3A shows that 86% of the aggressive basal-like tumors in these datasets are positively correlated with the HGF signature, whereas only 23.6% of the luminal A tumors present a positive association. Additionally, 40% of Her2-like tumors and 36% of luminal B tumors, both of which are more aggressive than luminal A tumors, were positively associated with the HGF signature (*P* <2.2×10^–16^). Furthermore, among basal-like patients that are positive for the HGF signature, patients had worse overall survival (Figure 3C). These results emphasize the importance of HGF signaling in aggressive breast cancer. While our coculture results show that HGF signaling is already present at the DCIS stage, the importance of this pathway in survival illustrates that the dysregulation of the HGF pathway persists in invasive basal-like tumors and contributes to their progression.

### Blocking of HGF inhibits three-dimensional morphogenesis

**Figure 4:**
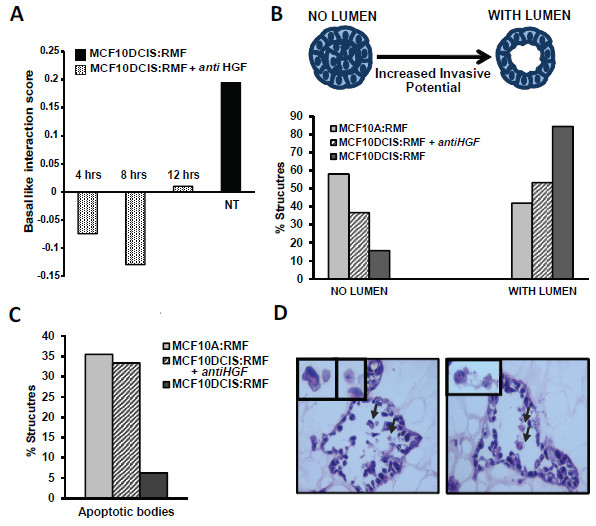
**Blocking hepatocyte growth factor signaling reverses basal-like microenvironments and slows morphogenesis in three-dimensional coculture. (A)** Basal-like interaction score of MCF10DCIS:RMF direct cocultures when treated with anti-hepatocyte growth factor (anti-HGF) antibody (as described in Materials and methods), despite the variability basal-like score being reversed from positive association to negative association. **(B)** Morphogenesis represents a balance between cell proliferation, apoptosis, and cellular migration. Morphogenesis assays track these processes *in vitro*. Bar graph shows the quantification of structures with and without lumens at 2 weeks in coculture. When HGF signaling is blocked in the ductal carcinoma *in situ* (DCIS) cocultures, the morphogenesis process is slowed down, causing these acinar structures to display an intermediate phenotype between the MCF10DCIS and the MCF10A cocultures. **(C)** Apoptosis was lowest for DCIS at 2 weeks, these cocultures had already undergone cavitation at that time point. Treatment with anti-HGF increases the apoptosis levels at this time-point because of delayed the cavitation process. **(D)** Representative hematoxylin and eosin images of cross-sections of the 3D structures and with apoptotic bodies. NT, not treated with anti HGF; RMF, reduction mammoplasty fibroblast.

Our previous studies of basal-like versus luminal cocultures indicated that hepatic fibrosis signaling was upregulated in basal-like cocultures [5]. In light of our current data illustrating that (1) MCF10DCIS:RMF cocultures have high basal-like interaction scores, (2) that HGF was secreted/active in MCF10DCIS cocultures, and (3) that HGF signaling is over-represented among invasive basal-like tumors and is prognostic of overall survival, we used HGF-targeted antibodies to study the role of HGF in the basal-like interaction score and functional morphogenic coculture assays. First, MCF10DCIS:RMF cocultures were incubated for 36 hours to allow for epithelial–stromal interactions to occur, and for the last 12 hours anti-HGF antibody was added every 2 hours. In Figure 4A we observe a decrease in the basal-like interaction score due to anti-HGF treatment. While there is variation (due to random or other unexplained causes) in the time-course data such that 4, 8 or 12 hours after exposure have basal-like interaction scores ranging from negative to slightly positive, it is clear that at all three time points the score is dramatically reduced.

Morphogenic assays have been shown to track normal, physiological acinar development as well as pathological malignant potential of epithelial cells [32]. The Brugge and Bissell laboratories have characterized the development of 3D structures over time in MCF10A cells, identifying important morphogenic mechanisms [33-35]. Namely, morphogenesis represents a balance between a variety of physiologic and pathologically-relevant processes including cell proliferation, apoptosis, and cellular migration [33]. We applied morphogenesis assays to develop an integrated picture of how key cellular phenotypes change with and without HGF signaling. Briefly, the expected development of MCF10A cells in 3D dictates that by 6 to 8 days they have reached their final size and will then start acquiring a lumen through apoptosis of the centrally located cells [36-38]. Using this as a metric, we performed 3D cocultures of MCF10A and MCF10DCIS with RMFs in a Matrigel®–collagen mix for 2 weeks. Cells were treated in the presence or absence of an anti-HGF antibody and the resulting acinar structures were analyzed using two techniques: longitudinal OCT imaging at two time points (1 week and 2 weeks), and traditional H&E and immunofluorescence staining at 2 weeks.

To track the morphogenesis of these cell lines over time, we used OCT because we have previously found that OCT imaging allows for longitudinal 3D *in vitro* imaging without disruption of the acinar structures [22]. In addition, with these images we can estimate the size of the acinar structures and their lumens. After counting 50 structures per condition, we observed no overall difference in the size of MCF10DCIS cocultures with and without anti-HGF antibody treatment. However, blocking HGF resulted in a statistically significant decrease in lumen size (*P* = 0.017, Figure 4B). The MCF10DCIS cocultures treated with the anti-HGF antibody bore a greater resemblance to the nonmalignant MCF10A (Additional file 6). As shown in Figure 4B, the number of structures without a lumen is high in MCF10A cocultures, and similar in the MCF10DCIS cocultures treated with anti-HGF, whereas the untreated MCF10DCIS cocultures have progressed to form a lumen (Additional file 7; *P* = 0.0007). These differences cannot be attributable to differences in proliferation rates of the cell lines, because the PDTs (in two-dimensional indirect cocultures) are very similar; PDTs of MCF10A and MCF10DCIS in coculture with RMF are 21.3 and 21.5 hours, respectively. When HGF signaling is blocked, these doubling times are 21.6 and 21.0 hours, respectively. The morphogenesis changes thus occur in the absence of statistically significant changes in proliferation (MCF10A *P* = 0.52 and MCF10DCIS *P* = 0.9).

Finally, H&E stains were used to measure apoptosis. By counting the presence of apoptotic bodies in the lumens, structures were classified as having apoptotic bodies or not [25]. Since apoptosis is the mechanism by which the lumens are formed [36-38], we would expect that in cells where lumen formation is not complete (MCF10A), the levels of apoptosis would be higher at a given time point. Figure 4C shows that, as expected, apoptosis was greater in the cocultures of MCF10A compared with MCF10DCIS cocultures and that treatment with anti-HGF antibody restores MCF10 apoptosis levels to those more similar to MCF10A (Additional file 8; *P* = 0.0450053). Taking these results collectively, treatment with anti-HGF reverts the MCF10DCIS cells to morphogenic phenotypes that resemble the less malignant cells (MCF10A), thereby blocking a microenvironment-mediated increase in malignant potential.

## Discussion

Breast cancer progression requires that epithelial cells acquire capabilities that enhance their growth and survival. While biological models of cancer have traditionally emphasized cell-autonomous characteristics, it is clear that changes in the microenvironment are also necessary [8]. Castro and colleagues demonstrated that the stroma of DCIS lesions already possess alterations found in full invasive tumors [39], and Ma and colleagues and Allinen and colleagues demonstrated that genomic changes occur in many cell populations of the microenvironment [10,11]. These models explicitly hypothesize that epithelial cells must undergo an evolutionary adaptation to their microenvironments [1,2,40]. Other models propose that the microenvironment is the driving force of the benign lesion evolution into full invasive tumors; Gatenby and Gillies even speculate that the origin of cancer may lie not in mutations within epithelial cells, but within acquired or somatic mutation changes in the mesenchymal cells that control tissue structure [41]. While our data do not support these latter models, our data do suggest that progression is not isolated to a single compartment (the epithelium), but rather reflective of epithelium–stroma coevolution. Heterotypic interactions between epithelium and stroma foster this coevolution by selecting for complementary phenotypes in stroma and epithelium.

Evidence of progressive complementarity between stroma and epithelium is critical to documenting coevolution. For example, considering transition from pre-invasive to invasive tumors, Hu and colleagues recently demonstrated that NF-κB and cyclooxygenase-2 signaling contributes to epithelial–stromal-mediated invasive potential [42]. In the current study we document coevolution in the earlier transition between benign and pre-invasive lesions. A striking upregulation of HGF was evident early in progression (at the atypical hyperplasia stage), but was not sufficient to induce the characteristic basal-like stromal-interaction phenotype. MET must also be expressed, and at the DCIS stage MET was upregulated with drastic consequences on the behavior of the epithelial cells: MCF10DCIS cells expressed high basal-like interaction scores and an associated ability to progress in morphogenesis assays. Similarly increased malignant potential has been observed in xenograft models with MCF10DCIS, which also preserve stromal interactions [43,44]. The importance of the HGF pathway was further documented in our system by blocking HGF signaling with an antibody, resulting in reversal of expression and morphologic phenotypes. In fact, this latter experimental work underscores an advantage of our *in vitro* coculture system: in studying cancer evolution using this cell line panel, pathways can be manipulated and direct causal effects can be examined. We were also able to separately assay both epithelial and stromal characteristics and show complementary changes. Epithelial cells upregulate the MET receptor only at the DCIS stage, despite HGF expression by all cocultured fibroblasts. While other studies have reported HGF expression by breast epithelial cells [45,46], these studies utilize different cell line models of progression and we did not observe a similar autocrine pathway of HGF–MET interaction in our studies. The previous studies focused on cell line models of metastatic progression, so perhaps our focus on pre-invasive stages of disease accounts for differences between findings. It is interesting to consider that by later stages of progression, this pathway may develop autocrine capability. By studying the interactions and the relative contributions of each component at different stages of progression, we can better understand how stroma and epithelium are coevolving.

Our results emphasize that HGF/MET signaling is important in basal-like breast cancer progression from early in the disease, but HGF has long been studied in invasive cancer biology and in normal development [47,48]. Previous studies have linked HGF/MET signaling with poor outcome in invasive breast cancers [49]. Several recent publications have demonstrated the importance of HGF/MET signaling and the microenvironment in melanoma treatment resistance [50,51]. Our data, using a novel HGF signature and three independent tumor datasets, indicate that HGF/MET signaling is highly correlated with basal-like breast cancer subtype and worse overall survival in patients. Mouse models with overexpression of MET induce basal-like tumors with signatures of WNT and epithelial to mesenchymal transition [52], suggesting that this pathway’s importance in tumor biology is conserved across species. In normal tissue, HGF is produced by stromal fibroblasts and acts as a mitogen, motogen and morphogen on MET-expressing epithelial cells [53]. MET is a tyrosine kinase receptor that, when activated by its ligand (HGF), auto-phosphorylates and initiates an intracellular signaling cascade that involves many targets. In the developing mammary duct, deletion of epithelial MET inhibits ductal branching [54]; and in adult glands, HGF is critical for tubulogenesis [55]. The HGF/MET pathway is thus an essential player in normal development and wound healing [56,57]. Given the high expression of wound response genes in the tissue adjacent to cancer [58] and the important role of HGF in normal ductal morphogenesis and invasive breast cancer, a better understanding of HGF/MET in progression of basal-like breast cancers is important.

Future work should focus on studying how molecules that block MET signaling affect stromal–epithelial interactions and should study HGF/MET in tissue from pre-invasive basal-like lesions. One case series reported by Lindemann and colleagues attempted to link HGF and MET signaling in earlier lesions by immunohistochemistry studies of HGF and MET in DCIS [59]. Their study concluded that an imbalance in MET expression between the tumor and the surrounding normal tissue is associated with aggressive DCIS phenotypes. However, uncertainty remains about how the imbalance can best be characterized in human tissue. Studying cell–cell communication is difficult in tissue, so the availability of complementary *in vitro* models is helpful. While these models rely on single cell lines and cannot represent the diversity of stromal phenotypes observed in humans, these coculture models advance our understanding of the reciprocal molecular changes in the pre-invasive stages of breast cancer and can guide research on tissue.

## Conclusion

Heterotypic interactions are crucial for disease progression; both compartments, the stroma and the epithelium, must coevolve to produce a successful tumor. Understanding the reciprocal epithelial and stromal changes that occur in early lesions will help to identify strategies to treat patients and/or prevent invasive breast cancers. HGF/MET signaling is a strong candidate pathway for treating premalignant basal-like lesions and the application of MET inhibitors should be considered in preclinical models to advance this plausible strategy.

## Consent

Written informed consent was obtained from the patients participating in this study under an Institutional Review Board-approved protocol.

## Abbreviations

3D: Three-dimensional; DCIS: Ductal carcinoma in situ; HAI-1: Hepatocyte growth factor activator inhibitor type 1; H&E: Hematoxylin and eosin; HGF: Hepatocyte growth factor; IPA: Ingenuity pathway analysis; OCT: Optical coherence tomography; PCR: Polymerase chain reaction; PDT: Population doubling time; RMF: Reduction mammary fibroblasts.

## Competing interests

The authors declare that they have no competing interests.

## Authors’ contributions

PC-H performed experiments, analyzed and interpreted data and drafted the manuscript. SMM, KM, KDA, JMF, LM, ER-P, RKC, ALO and HAB contributed to data collection and analysis and edited the manuscript. MD performed microarray analysis and drafted statistical methods. MAT conceived of the project, supervised experiments and analysis, and contributed to writing the final manuscript. All authors approved the manuscript.

## Supplementary Material

Additional file 1: Figure S1 Primary fibroblasts have temporal and intra-individual instability. mRNA quantification of HGF across a panel of 14 primary fibroblasts lines and the RMF cell line in coculture with the MCF10A progression series. Primary fibroblasts were isolated from five patients both from the cancer-adjacent tissue (CNAF: cancer-normal associated fibroblasts) and the tumor itself (CAF: cancer-associated-fibroblasts). HGF levels vary at the transcriptional level between patients and between passages. *Samples had no detectable levels of transcript by quantitative PCR. Click here for file

Additional file 2: Table S1 IPA pathway analysis and biological function analysis of genes upregulated in MCF10DCIS:RMF direct cocultures. Click here for file

Additional file 3: Figure S2 MCF10A series progressively acquires basal-like microenvironment characteristics also when in interaction cocultures. Basal-like interaction score of monocultures (M), indirect cocultures (I) and direct cocultures (D). Indirect cocultures maintain the trends of the basal-like interaction score present in the direct cocultures (from Figure 1B). Click here for file

Additional file 4: Table S2 List of cytokines detected on the antibody based array with the values for each coculture. Click here for file

Additional file 5: Figure S3 HGF is produced by the stromal component of the cocultures, the RMFs. **(A)** RNA levels of HGF (∆CT values relative to the 18s gene) in the monocultures of the MCF10A series and the RMFs, as well as these cell lines in coculture. The epithelial cells had no detectable levels of HGF transcript (*) even in coculture with RMFs; only RMFs had high levels of the transcript. **(B)** Protein levels of HGF, again of cells in monoculture or coculture. Epithelial cells in monoculture had no detectable levels of HGF (#); however in coculture they appear to have some HGF protein, and we argue this is due to the internalization of the receptor–ligand complex. RMFs had high levels of HGF expression both in monoculture and in coculture. Both graphs demonstrate the same trend, the stromal cells are responsible for HGF secretion in this coculture system. Click here for file

Additional file 6: Figure S4 OCT measurements of the acini structures. **(A)** Representative fluorescent pictures of acinar structures stained with pan-cytokeratin (green) and 4′,6-diamidino-2-phenylindole (DAPI; nucleus), the left picture shows a structure without a lumen and the right picture represent a structure with a very well-defined lumen. **(B)** Graphs representing the evolution of the overall size (area) of the acini and the size of the lumen (lumen). Anti-HGF treatment does not affect the overall size of the 3D structures; however, it has a big influence on the area of the lumen (*P = 0.017). Acini with anti-HGF treatment present smaller lumens resembling the more benign cell line MCF10A. Diagram adapted from [37], which shows the progression overtime of the different 3D cocultures that were performed. MCF10DCIS:RMF progress much faster through the morphogenesis assay than the MCF10A:RMF; MCF10DCIS:RMF depleted of HGF signaling present a phenotype similar to the less aggressive MCF10A. Click here for file

Additional file 7: Table S3 HGF signature: 280 genes that were upregulated (red) or downregulated (green) in the generated HGF signature. Click here for file

Additional file 8: Table S4 Chi-square analysis of 3D quantification of the morphological assay. Lumen and apoptosis quantification. Click here for file
