# Supplementary figures and images for: Role of HGF in epithelial–stromal cell interactions during progression from benign breast disease to ductal carcinoma in situ
Source: Breast Cancer Res. 2013 Sep 12;15(5):R82. doi: 10.1186/bcr3476 (PMC3978616; doi:10.1186/bcr3476)

Supplemental Figure 1

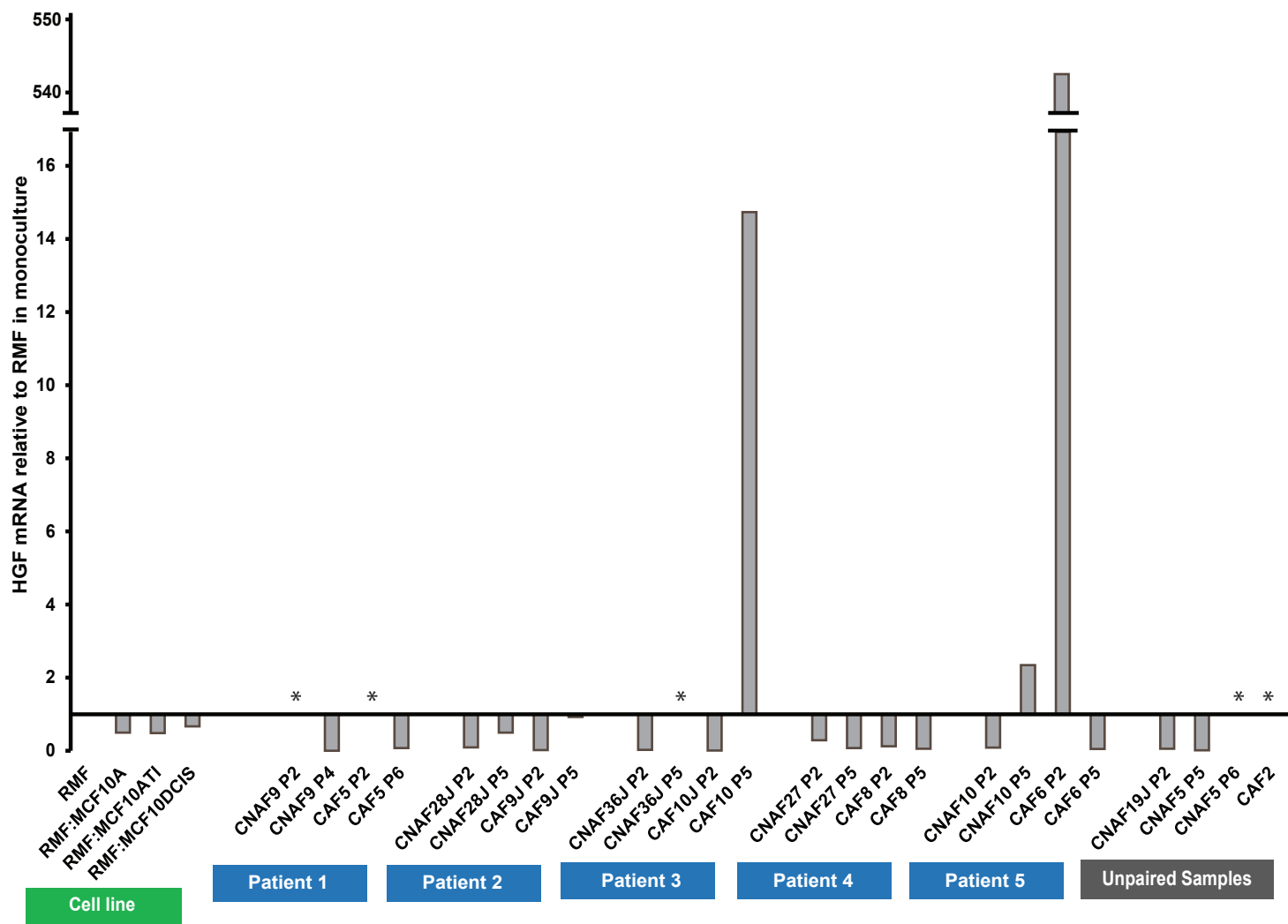

Supplement: Additional file 1: Figure S1 — Primary fibroblasts have temporal and intra-individual instability. mRNA quantification of HGF across a panel of 14 primary fibroblasts lines and the RMF cell line in coculture with the MCF10A progression series. Primary fibroblasts were isolated from five patients both from the cancer-adjacent tissue (CNAF: cancer-normal associated fibroblasts) and the tumor itself (CAF: cancer-associated-fibroblasts). HGF levels vary at the transcriptional level between patients and between passages. *Samples had no detectable levels of transcript by quantitative PCR. [file bcr3476-S1.pdf]

Supplemental Figure 2

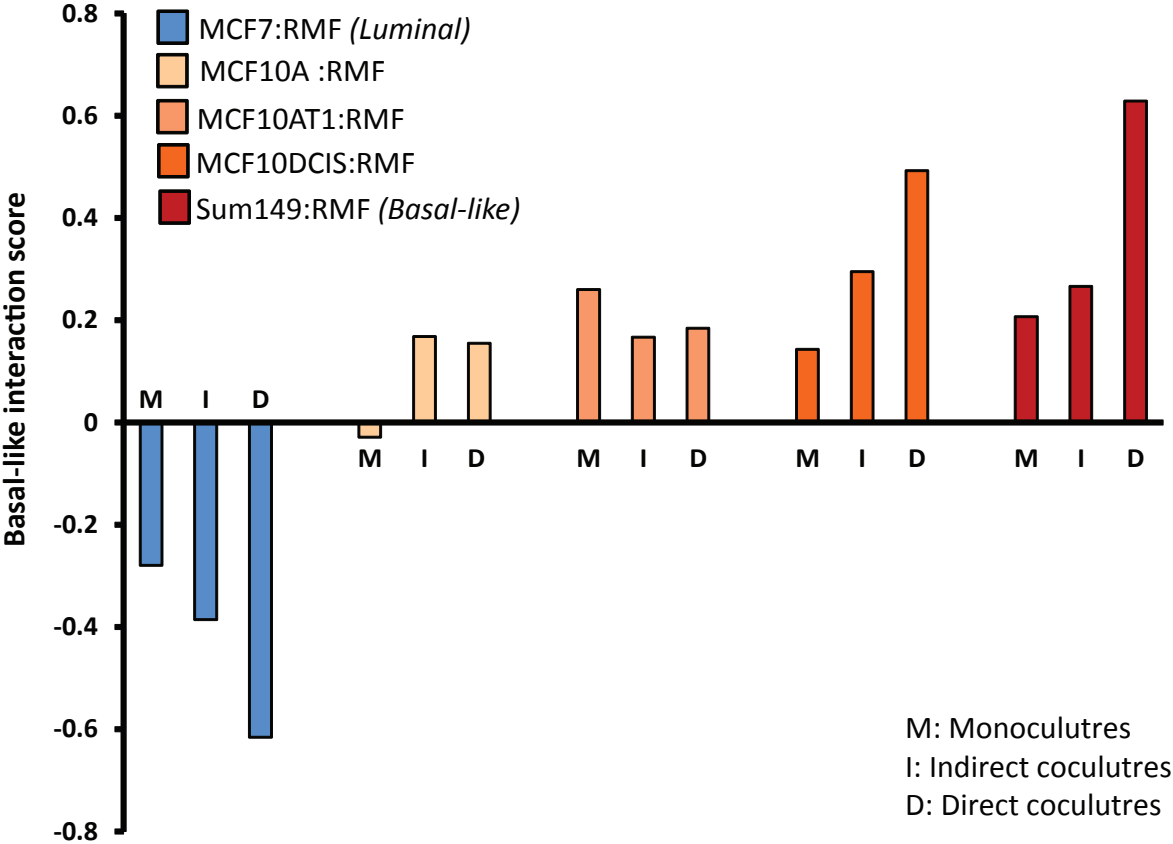

Supplement: Additional file 4: Table S2 — List of cytokines detected on the antibody based array with the values for each coculture. [file bcr3476-S4.pdf]

**A**

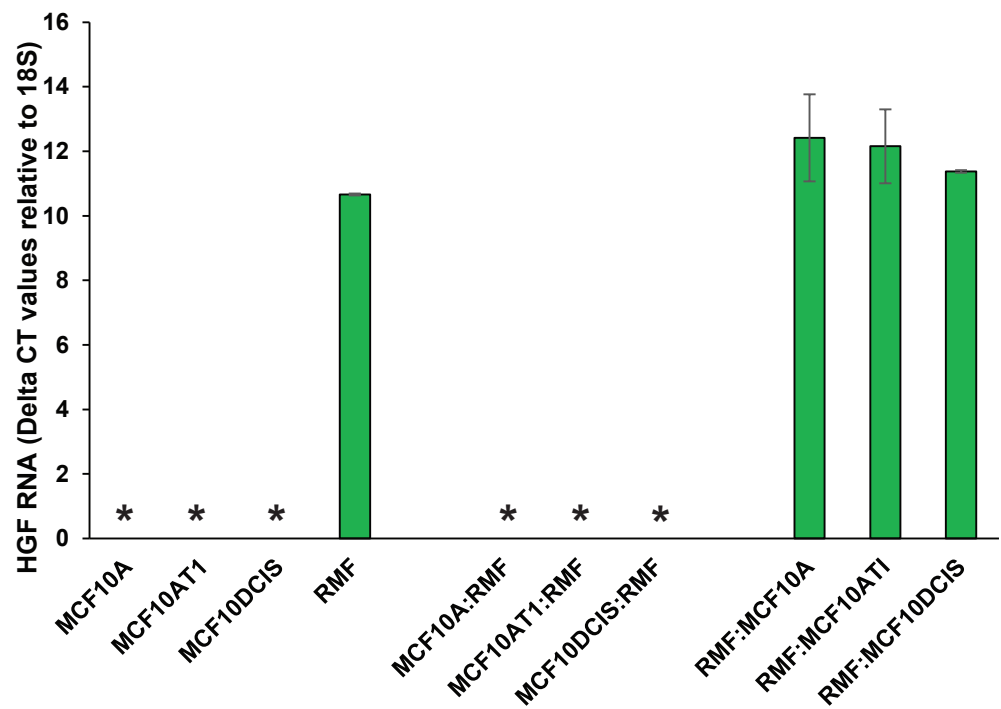

**B**

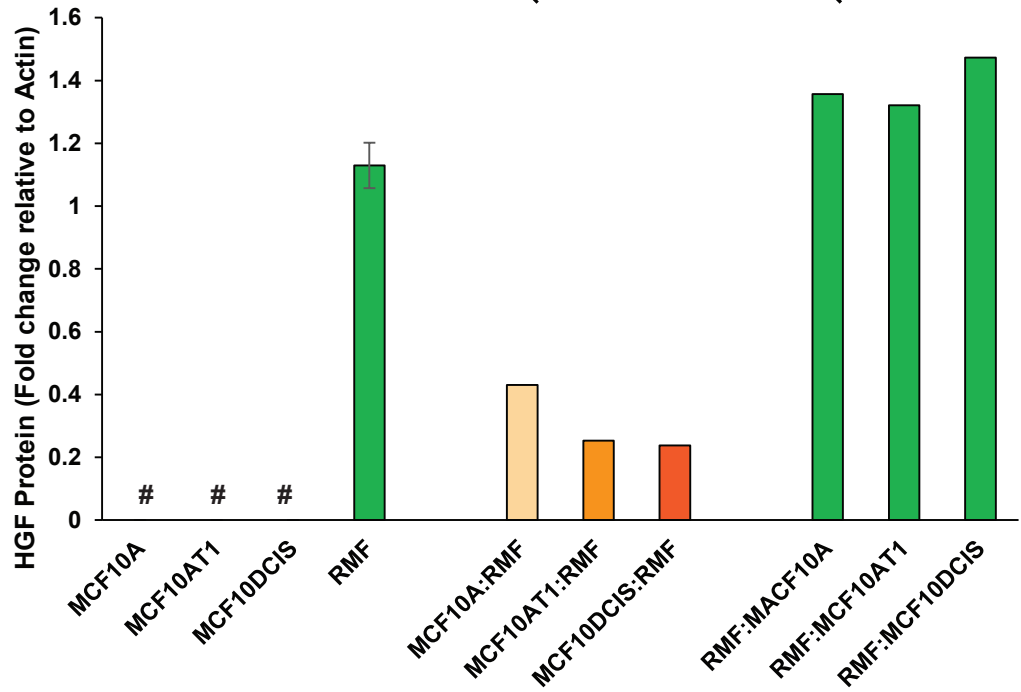

Supplement: Additional file 6: Figure S4 — OCT measurements of the acini structures. (A) Representative fluorescent pictures of acinar structures stained with pan-cytokeratin (green) and 4′,6-diamidino-2-phenylindole (DAPI; nucleus), the left picture shows a structure without a lumen and the right picture represent a structure with a very well-defined lumen. (B) Graphs representing the evolution of the overall size (area) of the acini and the size of the lumen (lumen). Anti-HGF treatment does not affect the overall size of the 3D structures; however, it has a big influence on the area of the lumen (*P = 0.017). Acini with anti-HGF treatment present smaller lumens resembling the more benign cell line MCF10A. Diagram adapted from [37], which shows the progression overtime of the different 3D cocultures that were performed. MCF10DCIS:RMF progress much faster through the morphogenesis assay than the MCF10A:RMF; MCF10DCIS:RMF depleted of HGF signaling present a phenotype similar to the less aggressive MCF10A. [file bcr3476-S6.pdf]

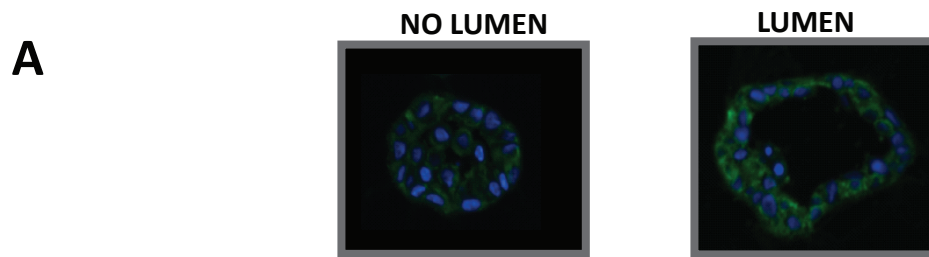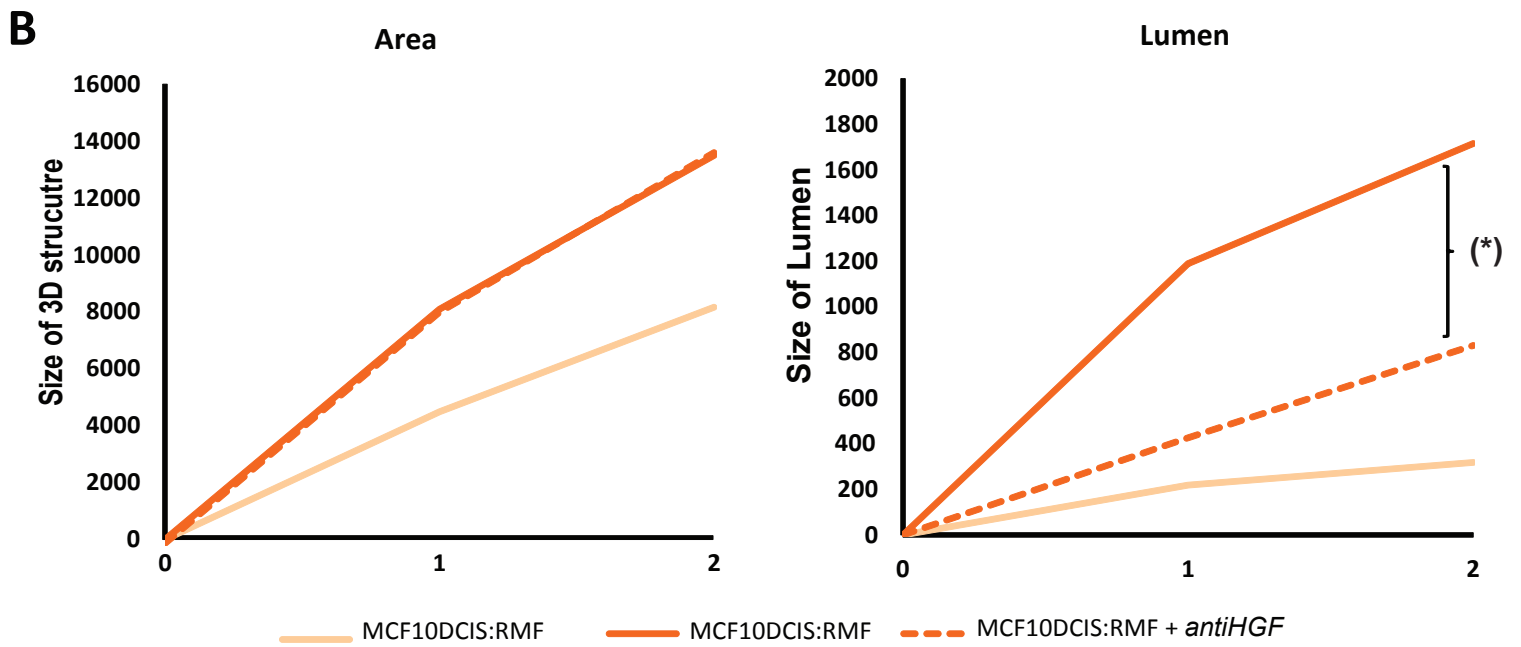

Supplement: Additional file 7: Table S3 — HGF signature: 280 genes that were upregulated (red) or downregulated (green) in the generated HGF signature. [file bcr3476-S7.pdf]
