# Supplementary material for: Role of HGF in epithelial–stromal cell interactions during progression from benign breast disease to ductal carcinoma in situ
Source: Breast Cancer Res. 2013 Sep 12;15(5):R82. doi: 10.1186/bcr3476 (PMC3978616; doi:10.1186/bcr3476)
Supplement: Additional file 3: Figure S2 — MCF10A series progressively acquires basal-like microenvironment characteristics also when in interaction cocultures. Basal-like interaction score of monocultures (M), indirect cocultures (I) and direct cocultures (D). Indirect cocultures maintain the trends of the basal-like interaction score present in the direct cocultures (from Figure 1B). [file bcr3476-S3.pdf]

Supplemental Table 2

	MCF10A:RMFs	MCF10AT1:RMFs	MCF10DCIS:RMFs
CKbeta8-1	-	-	1.57
MIP-3alpha	-	-	1.63
il-4	-	-	1.65
Eotaxin	-	-	1.66
TARC	-	-	1.68
Il-3	-	-	1.7
FGF-4	-	-	1.71
RANTES	3.13	2.32	1.8
BDNF	-	-	1.83
IGFBP-1	-	-	1.85
FGF-9	-	-	1.86
NAP-2	-	-	1.86
IL-13	-	-	1.95
Thrombopoetin	-	-	1.98
GDNF	-	-	2
leptin	-	-	2.01
BLC	-	-	2.02
Osteopontin	-	-	2.03
MDC	-	-	2.04
Eotaxin-3	-	-	2.05
MIP-1delta	-	-	2.06
PIGF	-	-	2.08
Fractalkine	-	-	2.09
SCF	-	-	2.11
Eotaxin-2	-	-	2.16
SDF-1	-	-	2.17
FGF-7	-	-	2.19
IGFBP-4	-	-	2.25
Il-1B	-	-	2.26
TNF-alpha	-	-	2.28
GM-CSF	-	-	2.3
LIF	-	-	2.32
Fit-3 ligand	-	-	2.33
MCSF	-	-	2.33
MIF	-	-	2.35
NT-3	-	-	2.35
IGF-1	-	-	2.35
IL-16	-	-	2.37
IGFBP-3	-	-	2.39
PARC	-	-	2.39
il-10	-	-	2.4
TGF-beta3	-	-	2.42
NT-4	-	-	2.48
LIGHT	-	-	2.5
il-5	-	-	2.51
MCP-4	-	-	2.55
il-7	-	-	2.66
I-309	-	-	2.66
TNF-beta	-	-	2.82
MIG	-	-	3.21
GCSF	-	-	3.59
Il-6	-	6.02	3.67
MCP-2	-	-	3.72
Il-2	-	-	3.8
FGF-6	-	1.56	5.04
GCP-2	-	3.18	5.58
MCP-3	-	2.5	6.09
Osteoprotegrin	-	-	6.21
ENA-78	-	2.37	6.24
MCP-1	-	7.98	7.57
Oncostatin M	-	-	11.14
HGF	-	70.12	80.65
