# Supplementary material for: Role of HGF in epithelial–stromal cell interactions during progression from benign breast disease to ductal carcinoma in situ
Source: Breast Cancer Res. 2013 Sep 12;15(5):R82. doi: 10.1186/bcr3476 (PMC3978616; doi:10.1186/bcr3476)
Supplement: Additional file 8: Table S4 — Chi-square analysis of 3D quantification of the morphological assay. Lumen and apoptosis quantification. [file bcr3476-S8.pdf]

Supplemental Table 4

	MCF10A:RMF	MCF10DCIS:RMF	MCF10DCIS:RMF *+antiHGF*	P-value
**Lumen**				
Yes	13	27	16	0.0007
No	18	5	11	
Undetermined	-	-	3	
**Apoptotic bodies**				
Yes	8	2	8	0.045
No	19	30	20	
Undetermined	3	-	2	
**Total (n)**	31	32	30	
